# Chili Pepper–Rice Rotation Alleviates Continuous-Cropping Constraints by Improving Nutrient Availability and Suppressing Pathogens via Rhizosphere Network Rewiring

**DOI:** 10.3390/plants15030400

**Published:** 2026-01-28

**Authors:** Rong Li, Ge Bai, Saifei Fan, Ying He, Jianhe Li, Zhaochen Wang, Bianhong Zhang, Yuanyuan Zhang, Xinyun Hu, Changxun Fang, Wenxiong Lin, Hongfei Chen

**Affiliations:** 1 College of Life Science, Fujian Agriculture and Forestry University, Fuzhou 350002, China; 18977408221@163.com (R.L.); 15840260039@163.com (G.B.); 18749260625@163.com (S.F.); 13882545078@163.com (Y.H.); 15759057132@163.com (J.L.); wangzhaochen0316@163.com (Z.W.); 15750826208@163.com (B.Z.); zyuanyuan259@163.com (Y.Z.); 17779151874@163.com (X.H.); 2 Fujian Key Laboratory of Agroecological Processing and Safety Monitoring, College of Agriculture, Fujian Agriculture and Forestry University, Fuzhou 350002, China; changfangxingyx@163.com (C.F.); lwx@fafu.edu.cn (W.L.); 3 Key Laboratory of Crop Ecology and Molecular Physiology, Fujian Province University, Fuzhou 350002, China

**Keywords:** chili pepper-rice rotation, continuous cropping obstacles, yield, rhizosphere microorganisms

## Abstract

Chili pepper (*Capsicum annuum* L.) is a globally significant economic crop, however long-term continuous cropping often induces multifaceted constraints including soil nutrient depletion, rhizosphere microbial imbalance, and pathogen accumulation, which collectively exacerbate soil-borne diseases and substantially reduce yield. Incorporating rice (*Oryza sativa* L.) into rotation increases the diversity of the cultivation environment and represents a cost-effective strategy to mitigate continuous-cropping obstacles. Therefore, evaluating and elucidating the role and underlying mechanisms of the chili pepper–rice rotation system in improving soil conditions and alleviating continuous cropping obstacles in chili pepper holds significant importance. This study conducted a two-year field experiment from 2023 to 2024, setting up chili pepper–rice rotation (RVR) and chili continuous cropping (CCV) treatments, to systematically analyze the effects of chili pepper–rice rotation on chili pepper yield, disease occurrence, soil nutrients, and rhizosphere microbial communities. Across 2023–2024, RVR significantly reduced the incidence of bacterial wilt and root rot, increasing yield by 10.60% in 2023 and by 61.07% in 2024 relative to CCV. Analysis of soil physicochemical properties revealed that RVR significantly promoted the accumulation of available nitrogen, phosphorus, and potassium in the soil, as well as enhanced nutrient-acquisition enzyme activity, effectively alleviating the carbon and phosphorus limitations faced by rhizosphere microorganisms. Rhizosphere microbial analysis indicated that under the RVR treatment, the abundance of pathogen-associated taxa such as *Ralstonia* and *Fusarium* significantly decreased. The co-occurrence network modularity increased, and the negative cohesion of pathogens was strengthened, thereby inhibiting pathogen expansion. Further random forest and correlation analyses demonstrated that RVR significantly contributed to yield formation by optimizing fungal metabolic pathways, such as galactose degradation, sulfate reduction, and L-tryptophan degradation. In conclusion, the chili pepper–rice rotation significantly alleviates continuous cropping obstacles and enhances yield by improving nutrient supply and regulating microbial community composition, as well as the topological structure and functional relationships of their co-occurrence networks, particularly by strengthening the role of fungi in community function and metabolic regulation. This study provides a theoretical basis for the biological and soil regulation of pepper continuous cropping obstacles and offers a feasible pathway for sustainable cultivation and green control strategies.

## 1. Introduction

Chili pepper (*Capsicum annuum* L.) is an annual or perennial plant of the Solanaceae family, possessing both vegetable and spice characteristics, making it an important economic crop [1]. In 2019, the global cultivation area spans approximately 1.99 million hectares, with an annual production of 38.02 million tons, and the market demand remains robust [2]. According to the 2022 statistics from the Food and Agriculture Organization (FAO), China leads global production with 16.57 million tons of fresh chili peppers [3]. Despite the stable and widespread cultivation of chili peppers, numerous limiting factors still hinder yield and quality improvements, including a lack of germplasm resources, frequent pest and disease outbreaks, climate change impacts, and the tightening of arable land resources [4,5]. To enhance land use efficiency, some major production areas have accelerated planting cycles and implemented high-frequency continuous cropping and multiple cropping systems in an effort to increase yield per unit area [6]. However, intensive cultivation practices, coupled with significant continuous cropping obstacles, have led to typical symptoms in plants, such as root rot, premature aging, and flower and fruit drop [7]. These issues result in a substantial decline in both yield and quality, thereby becoming the primary bottleneck restricting the sustainable and stable production of chili peppers [8].

Continuous cropping obstacle refers to a series of issues, including restricted crop growth, reduced yield, deteriorating quality, and increased pests and diseases, that arise when the same or similar types of crops are planted consecutively for several years in the same soil, even with the implementation of conventional cultivation management practices [9]. Existing studies commonly attribute continuous cropping obstacles to soil acidification, nutrient imbalance, and the accumulation of allelopathic substances [10]. However, considering that soil itself has a strong buffering capacity, short-term (e.g., within 2–3 cropping cycles) physicochemical changes caused by monoculture alone are insufficient to explain such significant yield reductions, suggesting that the mechanisms behind continuous cropping obstacles are far more complex than previously understood. As research deepens, the academic focus has shifted from soil physicochemical properties and the accumulation of allelopathic substances to biological processes. Increasing evidence suggests that changes in the rhizosphere microbial community are a key driver of continuous cropping obstacles [11]. The deterioration of soil physicochemical conditions, together with shifts in the rhizosphere environment, can markedly reduce microbial diversity [12], accompanied by declines in beneficial taxa (e.g., the phylum Actinobacteria and the genera *Pseudomonas* and *Bacillus*) and the accumulation of soil-borne pathogens, including fungal pathogens (e.g., *Fusarium* and *Rhizoctonia*) and fungal-like oomycetes (e.g., *Pythium*), ultimately increasing disease incidence and severity [13,14]. Consequently, the frequency and severity of diseases increase. The complexity and stability of the microbial community are reduced, the synergistic network structure is disrupted, and antagonistic mechanisms and ecological functions are impaired, thereby weakening the buffering and recovery capacity of the soil system [15,16]. The deep disturbance of the rhizosphere microecology prevents chili peppers from maintaining normal growth and yield, even under nutrient-sufficient conditions, reflecting the highly coupled relationship between soil, microorganisms, and plants. In this process, the feedback mechanism between plants, microorganisms, and soil may exacerbate the imbalance of the rhizosphere microecology, further limiting the growth and yield of chili peppers.

To mitigate the damage caused by continuous cropping obstacles, various control measures, including agricultural, physical, chemical, and biological approaches, have been adopted. Some of these measures, such as the application of bio-organic fertilizers, inoculation of antagonistic bacteria, and soil disinfection, can temporarily alleviate diseases [17]. However, their widespread adoption is often limited by high costs, low sustainability, and potential ecological risks [18]. In contrast, crop rotation, by introducing diversity in crop types and cultivation environments, effectively regulates soil microbial structure and disrupts the continuity of pathogen ecological niches, making it an important low-cost strategy for alleviating continuous cropping obstacles [19]. Previous studies have reported that rotation between grass crops and solanaceous vegetables promotes the recovery of beneficial microbial communities and inhibits pathogen accumulation through differences in root exudates and soil disturbance, significantly improving the crop growth environment [20]. Research on rice (*Oryza sativa* L.) paddy ecosystems as a platform for controlling continuous cropping obstacles has gradually increased. The long-term waterlogging and reductive environment during the rice growth period significantly alter soil redox conditions, reducing oxygen levels and effectively inhibiting the survival and reproduction of aerobic pathogens, such as *Fusarium* spp. and *Rhizoctonia* spp., thereby reducing disease risks [21]. Additionally, rice root exudates and their rhizosphere microbial communities exhibit a higher abundance of facultative anaerobic bacteria, in stark contrast to the aerobic microbial communities present in dryland soils. This environmental difference not only regulates soil microbial community structure but also influences soil nutrient cycling and metabolic functions, promoting the diversification and stabilization of ecosystem functions [22]. Previous studies have shown that crop rotation systems, such as tomato(*Solanum lycopersicum* L.)-rice and potato(*Solanum tuberosum* L.)-rice, significantly enhance the diversity and functional richness of rhizosphere microorganisms in subsequent crops [23,24]. These systems strengthen key functional microbial communities involved in nitrogen fixation, phosphorus solubilization, and pathogen antagonism, thus improving crop disease resistance and nutrient use efficiency. Most microecological evidence for crop rotation has been derived from Solanaceae crops such as tomato and potato; however, for the key diseases driving continuous-cropping obstacles in chili pepper (bacterial wilt and root rot), directly comparable and systematic evidence linking shifts in pathogen-associated taxa to the reconfiguration of rhizosphere interaction networks remains limited. Building on shared rhizosphere recruitment traits among Solanaceae crops and the strong perturbation of the soil microenvironment imposed by wet–dry transitions during the rice season, we focus on a chili pepper–rice rotation as a representative paddy–upland rotational system that aligns with seasonal farming schedules. Nevertheless, comprehensive assessments of how this system shapes rhizosphere community structure, functional diversity, and soil ecosystem stability in chili pepper remain scarce, and the underlying ecological regulatory mechanisms warrant further elucidation.

Based on this, the present study used chili continuous cropping as a control to investigate the effects of chili continuous cropping and chili pepper–rice rotation on soil physicochemical properties and rhizosphere microbial communities. It is hypothesized that chili–rice rotation can improve the soil environment, enhance rhizosphere microbial diversity and functional community abundance, inhibit pathogen expansion, and, thereby, alleviate continuous cropping obstacles. The study aims to systematically assess: (1) the effects of chili pepper–rice rotation on chili pepper yield, as well as the incidence of root rot and bacterial wilt; (2) the changes in soil environment, microbial community structure, and function under the rotation system; (3) the rhizosphere ecological mechanisms by which chili pepper–rice rotation alleviates chili pepper continuous cropping obstacles. The results of this research are expected to provide strong theoretical support for the ecological regulation of chili continuous cropping obstacles, deepen the understanding of soil microecological regulation mechanisms in “paddy–upland” rotation systems, and lay the foundation for the scientific advancement of the chili–rice rotation model. This study will contribute to the development of a green and efficient chili cultivation model that combines ecological and production benefits, promoting the transformation of chili agriculture towards sustainable development and achieving a harmonious balance between resource utilization and environmentally friendly agricultural practices.

## 2. Results

### 2.1. Effects of Different Cultivation Patterns on the Incidence and Yield of Chili Peppers

**Figure 1:**
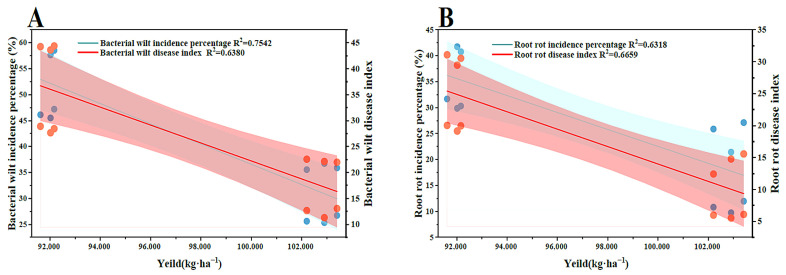
Linear regression analysis of yield and the incidence and disease severity index of bacterial wilt (**A**) and root rot (**B**). Red circles indicate the RVR treatment, and blue circles indicate the CCV treatment.

As shown in Table 1, across two consecutive years (2023–2024), the chili pepper–rice rotation (RVR) consistently reduced disease pressure from bacterial wilt and root rot while maintaining higher and more stable yield than chili continuous cropping (CCV). In 2023, relative to CCV, RVR decreased the incidence of bacterial wilt and root rot by 22.10% and 18.97%, respectively, and reduced the corresponding disease indices by 21.57% and 27.74%, accompanied by a 10.60% increase in yield. In 2024, disease severity under CCV markedly intensified (incidence: 58.49% for bacterial wilt and 40.91% for root rot; disease index: 44.08 and 30.36, respectively), and yield declined by 29.97% compared with 2023. In contrast, RVR maintained a yield comparable to that in 2023 and significantly alleviated both diseases in 2024; compared with CCV, RVR reduced bacterial wilt incidence and disease index by 55.72% and 73.48%, and reduced root rot incidence and disease index by 71.99% and 80.70%, respectively, resulting in a 61.07% higher yield (*p* < 0.05). Linear regression further indicated that yield was significantly negatively associated with both incidence and disease index of bacterial wilt (Figure 1A, R^2^ = 0.6380~0.7542) and root rot (Figure 1B, R^2^ = 0.6318~0.6659).

In summary, the two-year experiment confirmed that chili pepper–rice rotation significantly inhibited the occurrence of bacterial wilt and root rot, leading to a significant increase and stable maintenance of chili pepper yield.

### 2.2. Effects of Different Planting Patterns on Rhizosphere Soil Nutrients and Microbial Nutrient Acquisition Enzymes

**Figure 2:**
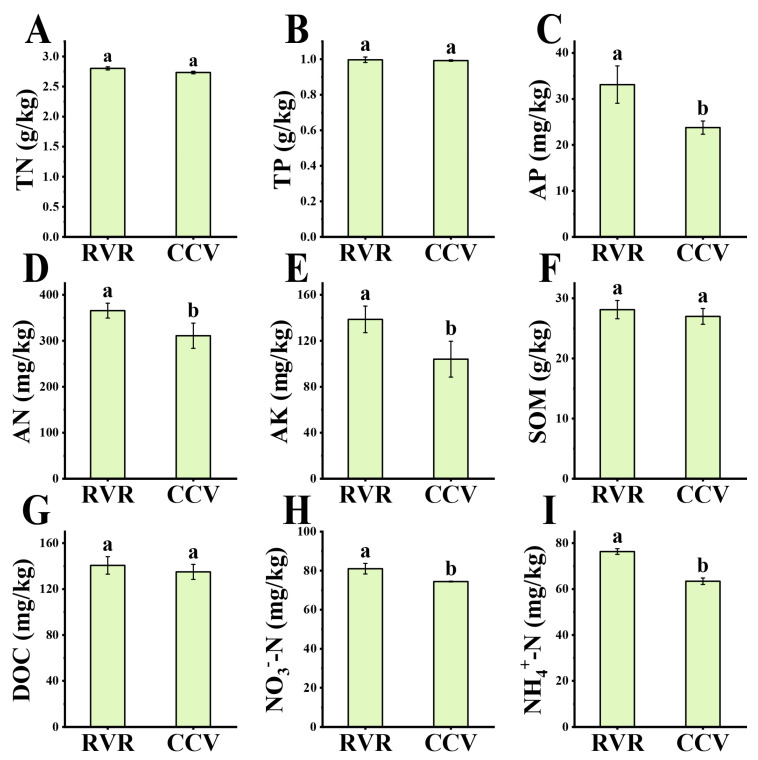
Rhizosphere soil nutrient properties under chili pepper–rice rotation (RVR) and chili continuous cropping (CCV). (**A**) TN, (**B**) TP, (**C**) AP, (**D**) AN, (**E**) AK, (**F**) SOM, (**G**) DOC, (**H**) NO_3_^−^-N, and (**I**) NH_4_^+^-N. Values are mean ± SD (n = 3). Different lowercase letters indicate significant differences between treatments (one-way ANOVA followed by Duncan’s multiple range test, *p* < 0.05).

As shown in Figure 2A,B, no significant differences were observed between the RVR and CCV treatments in terms of total nitrogen (TN), total phosphorus (TP), and soil organic matter (SOM) contents. Regarding available nutrients, compared to the CCV treatment, the RVR treatment significantly increased the levels of available phosphorus (AP), alkali-hydrolyzable nitrogen (AN), available potassium (AK), nitrate nitrogen (NO_3_^−^-N), and ammonium nitrogen (NH_4_^+^-N), with increases of 39.31%, 17.53%, 33.29%, 8.82%, and 20.36%, respectively. The dissolved organic carbon (DOC) content in the RVR treatment was slightly higher than that in the CCV treatment, but the difference was not statistically significant (Figure 2C–I).

**Figure 3:**
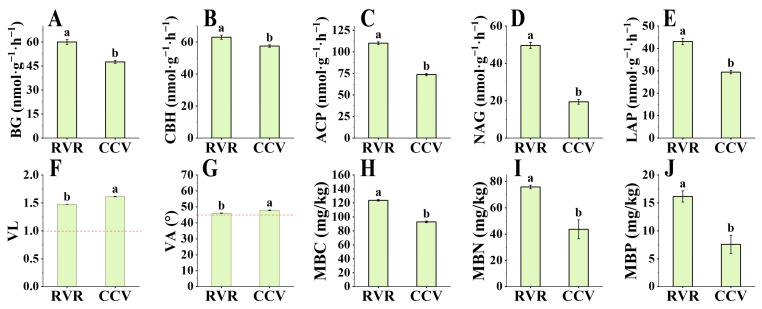
Rhizosphere ecoenzyme activities and microbial nutrient limitation under RVR and CCV. (**A**–**E**) Activities of BG, CBH, NAG, LAP, and ACP. (**F**,**G**) Vector analysis of ecoenzymes showing vector length (VL; microbial C limitation) and vector angle (VA; relative P vs. N limitation). (**H**–**J**) Microbial biomass C (MBC), microbial biomass N (MBN), and microbial biomass P (MBP). Values are mean ± SD (n = 3). Different lowercase letters indicate significant differences between treatments (one-way ANOVA followed by Duncan’s multiple range test, *p* < 0.05).

As shown in Figure 3, RVR significantly increased the activity of rhizosphere microbial enzymes associated with nutrient acquisition. Compared to the CCV treatment, the activities of β-glucosidase (BG), cellobiohydrolase (CBH), N-acetyl-β-glucosaminidase (NAG), leucine aminopeptidase (LAP), and acid phosphatase (ACP) were significantly higher in the RVR treatment (Figure 3A–E), with increases of 26.21%, 9.55%, 49.48%, 155.33%, and 49.36%, respectively. The vector model analysis of nutrient acquisition enzymes was employed to assess microbial nutrient limitation (Figure 3F,G). The results showed that, compared to CCV, RVR significantly reduced the vector length (VL) (*p* < 0.05), effectively alleviating the issue of soil carbon limitation. The vector angle (VA) of both RVR and CCV treatments was greater than 45°, indicating that phosphorus limitation was the dominant constraint for rhizosphere microorganisms. Meanwhile, the VA of the CCV treatment was significantly higher than that of the RVR treatment, indicating a more severe degree of phosphorus limitation for rhizosphere microorganisms under continuous cropping. In addition, the microbial biomass carbon (MBC), microbial biomass nitrogen (MBN), and microbial biomass phosphorus (MBP) in the RVR treatment were all significantly higher than those in the CCV treatment (*p* < 0.05, Figure 3H–J). In summary, the pepper–rice rotation system could alleviate carbon and phosphorus limitations simultaneously by enhancing the activity of nutrient-metabolizing enzymes in rhizosphere microorganisms, while also promoting the accumulation of microbial biomass.

### 2.3. Effects of Different Planting Patterns on Pathogen Abundance

High-throughput sequencing of the bacterial 16S rRNA V3–V4 region and fungal ITS region was conducted to profile rhizosphere microbial communities. The results of the community diversity analysis are presented in Table 2. The bacterial community diversity in the RVR treatment group showed a slight increase compared to the CCV treatment group, while the fungal community diversity exhibited a slight decrease. However, the differences in both bacterial and fungal community diversities between the two treatment groups were not statistically significant.

**Figure 4:**
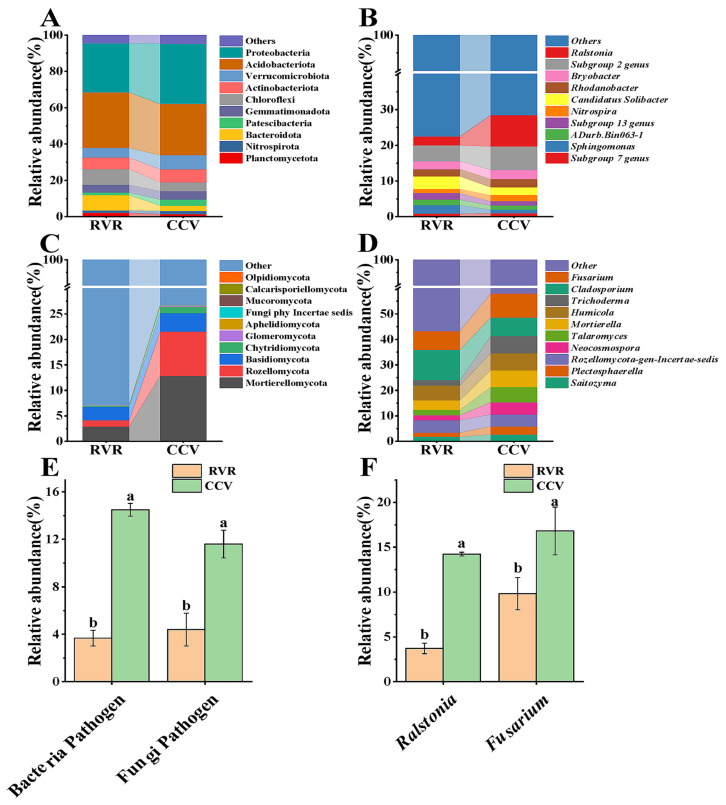
Taxonomic shifts in rhizosphere microbial communities under RVR and CCV. (**A**) Bacterial composition at the phylum level; (**B**) bacterial composition at the genus level; (**C**) fungal composition at the phylum level; (**D**) fungal composition at the genus level; (**E**) relative abundance of putative bacterial and fungal pathogens; (**F**) relative abundance of *Ralstonia* and *Fusarium*. Values are mean ± SD (n = 3). Different lowercase letters indicate significant differences between treatments (one-way ANOVA + Duncan’s test, *p* < 0.05).

Taxonomic analysis of the top 10 dominant phyla and genera (Figure 4) revealed notable shifts in community structure. At the phylum level, the RVR treatment significantly increased the relative abundance of Bacteridota and Chloroflexi (*p* < 0.05), while significantly decreasing that of Proteobacteria (Figure 4A). For fungi, at the phylum level, the relative abundance of Mortierellomycota and Rozellomycota was significantly lower in the RVR treatment compared to the CCV treatment (Figure 4C). At the genus level, the RVR treatment significantly enhanced the abundance of *Candidatus Solibacter* in the bacterial community (Figure 4B) and *Cladosporium* in the fungal community (Figure 4D). More importantly, RVR significantly suppressed the relative abundance of pathogen taxa (Figure 4E). For representative putative soil-borne pathogen-associated taxa (e.g., *Ralstonia* and *Fusarium*, annotated at the genus level), their relative abundances were significantly lower under RVR than under CCV (*p* < 0.05, Figure 4F). These findings suggest that crop rotation effectively reshapes the rhizosphere microbial community structure and reduces the proliferation of pathogens.

### 2.4. Characteristics of Pathogen Network Structure Changes Under Different Planting Patterns

**Figure 5:**
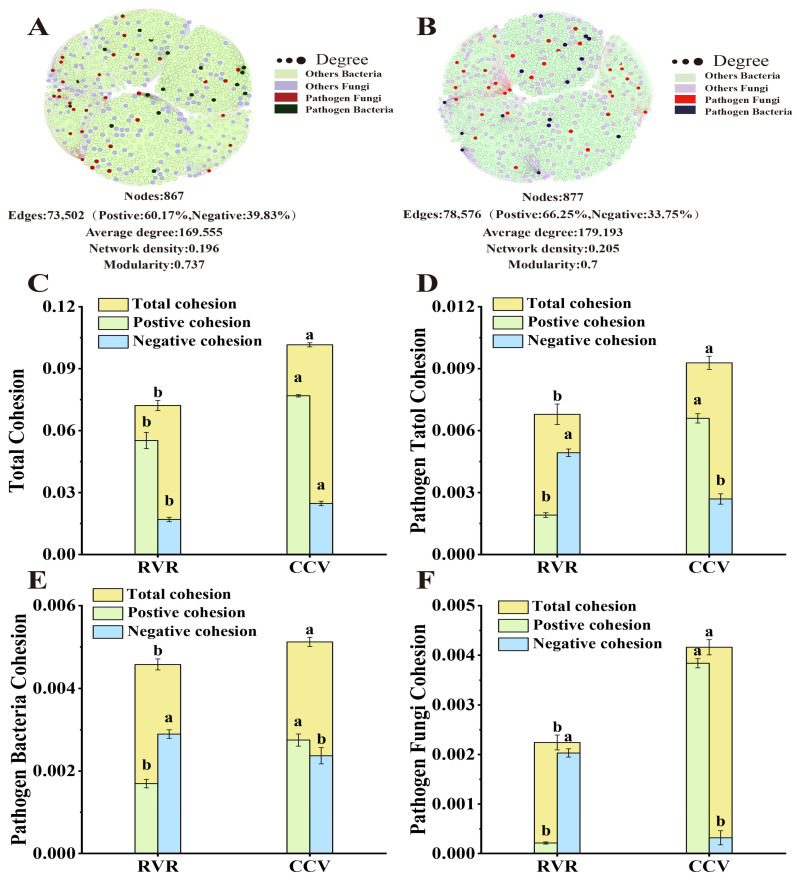
Co-occurrence network analysis of rhizosphere microbial communities under different planting patterns. (**A**): chili–rice rotation; (**B**): chili continuous cropping; treatment-specific networks (nodes represent ASVs; edges represent significant Spearman correlations retained under the stated threshold). (**C**): Community cohesion of RVR and CCV; (**D**): pathogen community cohesion of RVR and CCV; (**E**): pathogen bacteria community cohesion of RVR and CCV; (**F**): pathogen fungi community cohesion of RVR and CCV. Values are mean ± SD (n = 3). Different lowercase letters indicate significant differences (*p* < 0.05).

Microbial co-occurrence network analysis revealed differences in the rhizosphere microbial community network characteristics between the RVR and CCV treatments (Figure 5A,B). Compared with CCV treatment, the RVR treatment exhibited fewer network nodes, a lower average degree, and reduced network density, while the proportion of negative correlation edges increased. The modularity of the CCV network (0.700) was lower than that of the RVR network (0.737), indicating stronger inter-module connectivity and higher overall coupling under CCV.

Using weighted calculations based on interspecies relationships and species abundance, the overall community cohesion in the RVR treatment was significantly lower than that in CCV treatment *(p* < 0.05, Figure 5C). Analysis focusing on pathogenic microorganisms showed that the total and positive cohesion of the pathogen community were significantly lower in the RVR treatment compared to CCV treatment (*p* < 0.05, Figure 5D), while negative cohesion was significantly higher (*p* < 0.05). Specifically, pathogenic bacteria in the RVR treatment exhibited reduced total cohesion and positive cohesion (*p* < 0.05, Figure 5E), as well as increased negative cohesion (*p* < 0.05). Pathogenic fungi, in contrast, showed highly significant reductions in both total cohesion and positive cohesion (*p* < 0.05, Figure 5F) and a significant increase in negative cohesion (*p* < 0.05).

**Figure 6:**
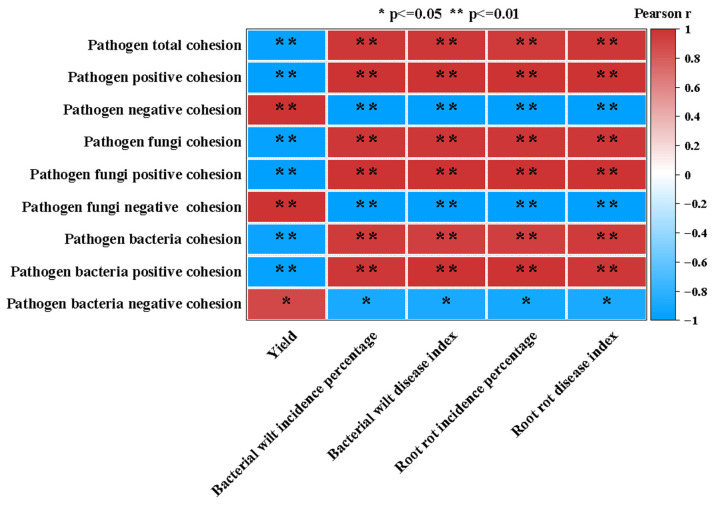
Correlations between yield/disease traits and microbial cohesion indices. Heatmap shows Pearson correlation coefficients; * and ** indicate significance at *p* < 0.05 and *p* < 0.01 (two-tailed), respectively.

Pearson correlation analysis (Figure 6) showed that the yield of chili peppers was significantly positively correlated with the negative cohesion of total pathogens, pathogenic fungi, and pathogenic bacteria (*p* < 0.05). Disease incidence and disease index were significantly positively correlated with the positive cohesion of pathogens (*p* < 0.05), whereas bacterial wilt incidence and disease index were significantly negatively correlated with the negative cohesion of pathogens (*p* < 0.05).

### 2.5. Characteristics of Microbial Functional Changes Under Different Planting Patterns

**Figure 7:**
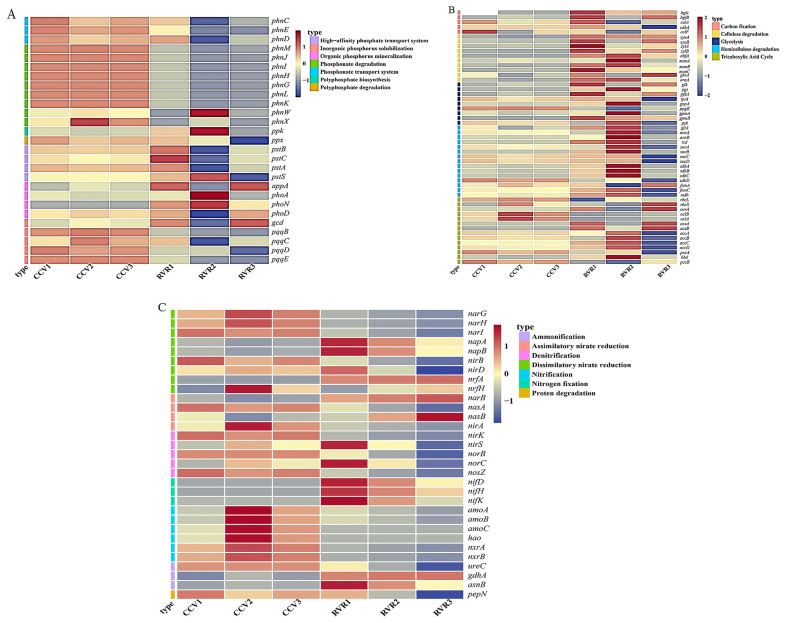
Functional gene profiles related to carbon (**A**), nitrogen (**B**), and phosphorus (**C**) cycles in the bacterial community.

**Figure 8:**
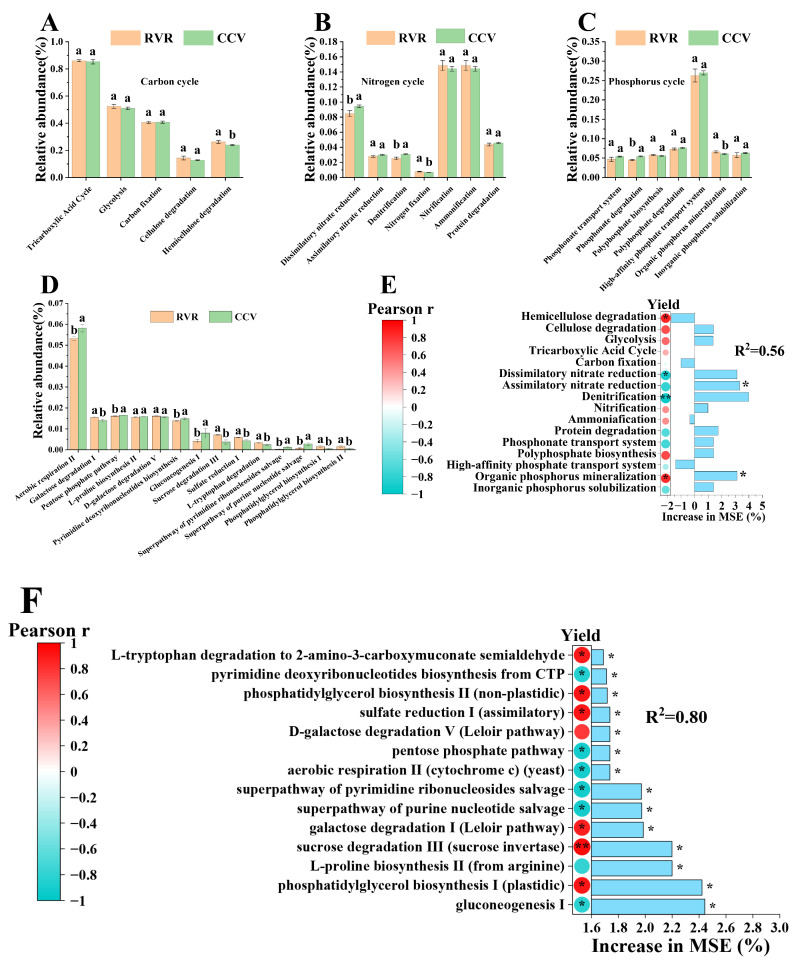
Functional features linked to yield under RVR and CCV. (**A**–**C**) Predicted bacterial genes related to C, N, and P cycling; (**D**) predicted fungal MetaCyc pathway profiles; (**E**,**F**) relationships between yield and bacterial C/N/P functions (**E**) and fungal metabolic pathways (**F**) identified by random forest and evaluated by Pearson correlation. Values are mean ± SD (n = 3). Different lowercase letters indicate significant differences (ANOVA + Duncan’s test, *p* < 0.05). * and ** indicate *p* < 0.05 and *p* < 0.01 (two-tailed), respectively.

To elucidate the functional changes in rhizosphere microbiome, bacterial genes involved in the carbon (C), nitrogen (N), and phosphorus (P) cycles were annotated using the KEGG database (Figure 7). In the carbon cycle pathway, the RVR treatment significantly increased the abundance of functional genes associated with hemicellulose degradation (*p* < 0.05, Figure 8A). In the nitrogen cycle pathway, the RVR treatment significantly reduced the abundance of functional genes involved in dissimilatory nitrate reduction and denitrification (*p* < 0.05), while significantly increasing the expression level of nitrogen fixation genes (*p* < 0.05, Figure 8B), suggesting a possible shift in the potential functional composition of N-transformation processes. In the phosphorus cycle pathway, the RVR treatment significantly enhanced the expression level of genes involved in organic phosphorus mineralization (*p* < 0.05, Figure 8C), while significantly decreasing the abundance of functional genes related to phosphonate degradation (*p* < 0.05).

Analysis of the relative abundance of fungal metabolic pathways based on the MetaCyc database (Figure 8D) revealed that pathways such as aerobic respiration II and pyrimidine deoxyribonucleotides biosynthesis were significantly more abundant in the CCV treatment than in the RVR treatment (*p* < 0.05). In contrast, pathways including galactose degradation I, sulfate reduction I, and L-tryptophan degradation were significantly more abundant in the RVR treatment compared to the CCV treatment (*p* < 0.05).

A random-forest regression model coupled with Pearson correlation analysis (Figure 8E,F) was used to evaluate the statistical associations between predicted functions and chili pepper yield. The results indicated that bacterial genes involved in C, N and P cycling contributed some predictive information in the model, yet only a limited subset showed significant correlations with yield: genes related to organic P mineralization were positively associated with yield (*p* < 0.05), whereas genes associated with dissimilatory nitrate reduction and denitrification were negatively associated with yield (*p* < 0.05). By contrast, the associations between fungal metabolic pathways and yield were more coherent: sucrose degradation and L-tryptophan degradation were positively correlated with yield (*p* < 0.05), whereas the pentose phosphate pathway, isopentenol biosynthesis, and aerobic respiration pathways were negatively correlated with yield (*p* < 0.05). Overall, the yield-associated signals from bacterial-predicted functions were relatively diffuse, whereas fungal metabolic pathways showed tighter statistical associations with yield.

### 2.6. Correlation Analysis of Key Factors in Pepper Growth Regulation Under Different Planting Systems

**Figure 9:**
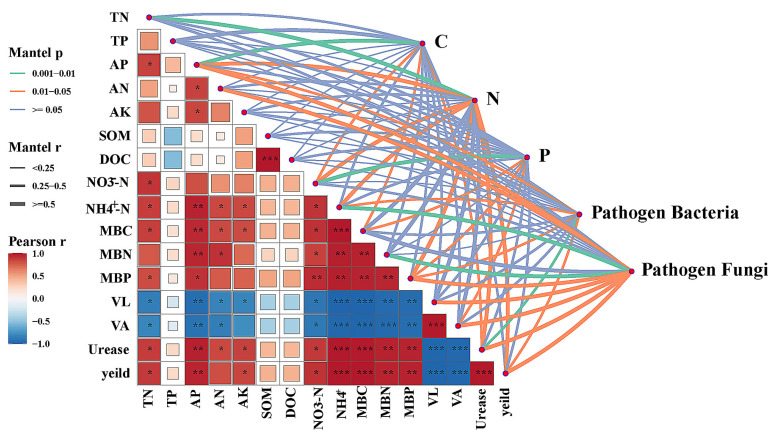
The relationships between functional genes of carbon, nitrogen, and phosphorus cycles, pathogen fungi, pathogen bacteria, and soil nutrients, microbial nutrient limitations, and pepper yield. Mantel links summarize multivariate associations, and pairwise Pearson correlations indicate trait-to-trait relationships.*, **, and *** indicate significance at *p* < 0.05, *p* < 0.01, and *p* < 0.001 (two-tailed), respectively.

The Mantel test, combined with Pearson correlation analysis, further clarified the associations among pathogen fungi, pathogen bacteria, carbon, nitrogen, and phosphorus cycling functional genes, soil nutrients, microbial nutrient limitations, and pepper yield (Figure 9). Pathogen bacteria were significantly correlated with AP, NH_4_^+^-N, MBP, urease activity, and pepper yield (*p* < 0.05). Pathogen fungi were significantly correlated with AN, AP, NH_4_^+^-N, MBN, and yield (*p* < 0.05). Meanwhile, pepper yield showed a strong positive correlation with MBC, MBN, and MBP (*p* < 0.01), but a significant negative correlation with pathogen fungi and bacteria. Additionally, carbon-cycling genes exhibited an extremely significant positive correlation with AP (*p* < 0.01), and were also significantly correlated with NO_3_^−^-N, MBC, and urease activity (*p* < 0.05). Nitrogen-cycling genes were significantly correlated with TN, AP, NO_3_^−^-N, NH_4_^+^-N, MBP, urease activity, and yield (*p* < 0.05), playing a key regulatory role in soil nitrogen turnover and yield formation. Phosphorus-cycling genes showed an extremely positive correlation with NO_3_^−^-N (*p* < 0.01) and were significantly correlated with MBC, MBP, and VA (*p* < 0.05). The Mantel test further confirmed that pepper yield was closely associated with the overall microbial network, highlighting the synergistic coupling among microbial functional potential, soil health, and crop growth performance.

## 3. Discussion

In this study, a two-year field experiment involving pepper–rice “paddy–upland” rotation (RVR) significantly increased pepper yield and reduced disease incidence (Table 1), consistent with the findings of Chen et al. [7]. The RVR treatment notably enhanced the levels of available nutrients, including AP, AN, AK, NO_3_^−^-N, and NH_4_^+^-N (Figure 2), in line with previous studies [25]. Soil nutrients not only serve as the fundamental material basis for crop growth but also act as key drivers of microbial community structure and functional differentiation [26]. Consistent with the improved nutrient status, RVR significantly increased the activities of ecoenzymes involved in C, N and P acquisition (Figure 3A–E); shifts in these nutrient-acquiring enzyme activities may reflect adjustments in rhizosphere microbial resource-acquisition strategies and associated functional processes [27]. Because extracellular enzyme activities are jointly shaped by microbial biomass, community composition and substrate conditions, differences in enzymes such as BG and NAG are more likely to represent an integrated microbial metabolic response to shifts in rhizosphere resource availability [28]. In parallel, by improving nutrient supply and cycling, RVR likely provided a more favorable resource context to sustain and modulate rhizosphere microbial metabolic activity.

Venter et al. [29] proposed that adequate nutrients and high microbial metabolic activity directly regulate rhizosphere community structure and diversity. The core issue of continuous cropping obstacles lies in the profound imbalance in the rhizosphere microbial community structure and its associated functions, and alterations in community structure driving the reconstruction of interaction networks [30]. In this study, high-throughput sequencing revealed that the RVR treatment had no significant effect on microbial α-diversity (Table 2), which contrasts with the conventional view that crop rotation generally enhances microbial diversity [31]. This inconsistency may be attributable to the long-term cultivation history of the experimental soil, which likely maintained a relatively high baseline diversity and thereby reduced the detectability of α-diversity shifts over the two-year period [32]. Against this background, the influence of RVR on the rhizosphere microbiota may preferentially manifest as selective responses of functional groups, thereby altering the functional organization of the community [33]. Specifically, the relative abundances of key microbial phyla involved in organic matter degradation and nutrient cycling, such as Bacteridota, Chloroflexi, Proteobacteria, and Mortierellomycota, exhibited significant changes under RVR treatment (Figure 4A–E), consistent with the observed variations in available nutrients and extracellular enzyme activity. Recent studies on multiple crops have demonstrated a strong association between the incidence of continuous cropping disorders and dynamic changes in soil microbial communities [34,35]. It should also be emphasized that the expansion of pathogen-associated taxa is typically driven by multiple, interacting factors (e.g., pH, moisture, organic matter, antagonistic microbiota and host susceptibility), rather than by any single determinant [35,36,37]. Moreover, environmental shifts commonly associated with continuous cropping, such as soil acidification, have been reported to promote the proliferation of *Ralstonia solanacearum* and increase the risk of bacterial wilt [38]. In our study, RVR significantly reduced the abundances of pathogen-associated bacterial and fungal taxa (Figure 4E,F), consistent with the sustained decline in *Fusarium* reported by Panneerselvam et al. following rice–legume rotation [39].

The decline in pathogen-associated taxa in chili pepper soils under RVR may reflect the joint action of multiple processes. First, flooding during the rice season can create anaerobic conditions that suppress aerobic pathogens [40]; drainage before harvest induces a wet–dry transition that may further alter the rhizosphere microenvironment, thereby reshaping microbial interactions and limiting the persistence of pathogen-associated taxa in continuous-cropping systems. Second, the rhizosphere microbiota may intensify biotic competition—through competition for siderophores, nutrients and ecological niches—thereby indirectly constraining the colonization advantage of pathogen-associated taxa [41]. Consistent with this, co-occurrence network analysis showed that RVR increased network modularity and strengthened negative correlations among taxa (Figure 5A,B), suggesting weakened positive co-occurrence among pathogen-associated taxa and relatively reduced inter-module connectivity. This pattern aligns with reports by Li et al. [42] in potato rotation, where higher modularity coincided with lower potential pathogenic fungi, and by Dasgupta et al. [43] under organic management, where modularity exceeded that under conventional management alongside reduced pathogen abundance. Further analysis showed that, relative to CCV, RVR reduced pathogen abundance by 62.03–74.62% and increased negative cohesion among pathogens by 83.23%. Previous studies suggest that more highly modular network architectures are often accompanied by a “compartmentalization” of interactions, which can help maintain community stability and reinforce competitive structuring [44,45], consistent with the significantly increased negative cohesion among pathogens under the RVR treatment (Figure 5D). Ning et al. [46] proposed that network modules can be viewed as a structural manifestation of microbial niche differentiation. In more highly modular networks, taxa tend to aggregate within relatively independent interaction units with fewer inter-module links; such “compartmentalization” may weaken synergistic co-occurrence among putative pathogen-associated taxa and between these taxa and the rest of the community, thereby reducing their potential to establish cross-module advantages.

Consistent with our functional predictions, bacterial genes associated with C, N and P cycling differed significantly between RVR and CCV in only a small number of modules (Figure 8A–C), and the subset showing significant correlations with yield was relatively limited, suggesting that bacterial functional potential may influence yield primarily through indirect pathways such as nutrient turnover and disease pressure (Figure 8E) [47]. By contrast, shifts in fungal metabolic pathways were more tightly associated with yield increases (Figure 8F), and thus may provide a more informative functional signal for yield variation [48,49]. Regarding carbon-source utilization, Wen et al. [50] reported in a tomato system that high pathogen pressure can reduce readily available rhizosphere carbon sources (e.g., sugars) and intensify resource competition. In a similar context, we observed higher relative abundances of aerobic respiration II and pyrimidine deoxyribonucleotide biosynthesis under CCV (Figure 8D), suggesting increased demand for readily available rhizosphere carbon and potentially intensified carbon competition under continuous cropping. In contrast, under RVR, pathways including galactose degradation I, sulfate reduction I and L-tryptophan degradation were significantly associated with yield (Figure 8F). The ecological implications of these pathways may instead reflect rotation-induced shifts in rhizosphere carbon transformation and redox-related processes, occurring alongside reduced pathogen pressure and improved host performance. For example, sulfate reduction under low-oxygen conditions may generate metabolites with antimicrobial activity [51,52], whereas tryptophan-related metabolism has been linked to plant growth and stress responses [53,54]. Notably, these interpretations are based on the observed differences and statistical associations in this study, together with evidence from the literature, and further experiments are required to validate the underlying causal pathways. Overall, Mantel tests and correlation analyses (Figure 9) showed that the relative abundances of pathogen-associated bacterial and fungal taxa were significantly associated with yield, indicating that the yield-promoting effect of chili pepper–rice rotation may be jointly related to reduced pathogen pressure and a reorganization of microbial functional potential, with fungal metabolic shifts showing the closest association with yield. Given that this was a two-year, single-site field study, future work should validate the findings across multiple sites and years and incorporate more process-based studies.

## 4. Materials and Methods

### 4.1. Experimental Site Overview

A two-year field experiment (2023–2024) was conducted in Dongsheng Village, Fuqing City, Fuzhou, Fujian Province, China (119.32° E, 25.82° N). The region has a subtropical monsoon climate with distinct seasons and abundant rainfall, and the soil type is clay loam. In 2023, the effective accumulated temperature and total precipitation during the chili pepper growing season were 4130.50 °C and 445.00 mm, respectively, while those during the rice growing season were 3351.50 °C and 933.33 mm. In 2024, the effective accumulated temperature and total precipitation during the chili pepper growing season were 4246.50 °C and 353.70 mm, respectively, while those during the rice growing season were 3352.00 °C and 1057.84 mm. Prior to trial establishment (0–20 cm), baseline soil properties were pH 5.66, soil organic matter (SOM) 28.65 g·kg^−1^, total nitrogen (TN) 2.76 g·kg^−1^, total phosphorus (TP) 0.98 g·kg^−1^, alkali-hydrolyzable nitrogen (AN) 336.37 mg·kg^−1^, available phosphorus (AP) 30.10 mg·kg^−1^, and available potassium (AK) 120.06 mg·kg^−1^.

### 4.2. Experimental Design

The experiment used the chili pepper cultivar “Niujiao 37–39” and the rice cultivar “Yongyou 4949” as test materials. A two-year field experiment (2023–2024) was established with two cropping systems: chili pepper–rice rotation (RVR) and chili continuous cropping (CCV), each with three replicate plots (100 m^2^ per plot; six plots in total). Within each year, pepper agronomic management (cultivar, transplanting date, planting density, irrigation, fertilization, and pest control) was kept identical between treatments; the only difference was the management of the preceding season (rice vs. fallow). During the rice-season window, CCV plots were maintained as clean fallow. After the preceding pepper harvest, aboveground crop residues were removed. Volunteer plants and weeds were controlled by manual removal and periodic shallow tillage at regular intervals, and no herbicide was applied. The fallow soil was kept non-flooded and was naturally wetted by rainfall (with no standing water); only routine field drainage was maintained to avoid waterlogging. This ensured that the only intended difference between treatments during the rice-season window was flooded rice cultivation (RVR) versus clean fallow (CCV).

Chili pepper seedlings were transplanted in September each year and harvested from November to April of the following year. Annual pepper yield was calculated as the cumulative fresh fruit yield across all harvests. In the RVR treatment, rice was cultivated under conventional paddy management, including flooding during key growth stages and drainage prior to harvest, and was managed following local high-yield practices. Rice fertilization followed local recommendations at 150 kg N·ha^−1^, 75 kg P_2_O_5_·ha^−1^, and 150 kg K_2_O·ha^−1^, supplied as urea (46% N), calcium superphosphate (12% P_2_O_5_), and potassium chloride (60% K_2_O) (Shanxi Shenhua Coal Chemical Group Co., Ltd., Shanghai, China). After rice harvest, fields were drained and prepared for pepper planting according to local practice (tillage and ridge preparation). In the CCV treatment, fields remained fallow during the rice season and were managed to minimize volunteer plants; plots were then prepared for pepper planting using the same tillage and ridge preparation procedures as in the RVR treatment.

### 4.3. Measurement Items and Methods

#### 4.3.1. Chili Pepper Yield and Disease Survey

At the chili pepper harvest stage, the fresh chili yield was measured based on the maturity of the chili peppers in each plot. The yield of fresh chili peppers was calculated using the weight obtained from multiple harvests [55].

At the harvest stage, the number of diseased plants in each treatment was recorded. The severity of bacterial wilt was rated on a 5-point scale according to the following criteria [56]: 0 = asymptomatic; 1 = mild symptoms, with leaf wilting less than 20%; 2 = moderate symptoms, with leaf wilting between 20% and 50%; 3 = severe symptoms, with leaf wilting between 50% and 80%; 4 = dead plant. The severity of root rot in chili peppers was assessed using a 0~7 scale, as described by Cai Gaolei et al. [57]: 0 = no lesions at the stem base; 1 = lesions around the stem base covering less than 25%; 3 = lesions around the stem base covering 25%~50%; 5 = lesions around the stem base covering 51%~75%, with plant wilting; 7 = lesions around the stem base covering more than 76%, with wilting, near death or dead plants.

Incidence rate = (number of diseased plants/total number of plants surveyed) × 100%

Disease index = [Σ (number of plants at each disease level × corresponding disease level value)/(total number of plants surveyed × highest disease level value)] × 100

#### 4.3.2. Rhizosphere Soil Sampling and Physicochemical Property Analysis

Rhizosphere soil was collected at the fruiting stage of chili pepper. In each plot, five uniformly growing plants were selected and carefully excavated with the entire root system. Loosely adhering soil was gently shaken off, and the remaining rhizosphere soil was pooled and homogenized to form one composite sample per plot. One subsample of fresh soil was stored at 4 °C for the determination of NH_4_^+^-N, NO_3_^−^-N, DOC, MBC, MBN, MBP, and enzyme activities. A second subsample was air-dried for the analysis of TN, TP, AP, AK, and SOM. A third subsample was flash-frozen in liquid nitrogen and stored at −80 °C for DNA extraction.

Total nitrogen (TN) and total phosphorus (TP) were determined using the HClO_4_-H_2_SO_4_ digestion method with a Smartchem 2000 automatic discrete chemical analyzer (Alliance Instruments, Italy) [58]. Ammonium nitrogen (NH_4_^+^-N) was quantified using the indophenol blue colorimetric method, while nitrate nitrogen (NO_3_^−^-N) was determined via the dual-wavelength spectrophotometric method. Available phosphorus (AP) was measured using the molybdenum–antimony anti-colorimetric method, and available potassium (AK) was determined by the ammonium acetate extraction-flame photometry method [59]. Dissolved organic carbon (DOC) was extracted using the cold water extraction method [59]. Microbial biomass carbon (MBC) was determined using the chloroform fumigation-K_2_SO_4_ extraction method, microbial biomass nitrogen (MBN) was measured using the chloroform fumigation extraction method, and microbial biomass phosphorus (MBP) was quantified using the chloroform fumigation extraction method [58].

The activities of β-1,4-glucosidase (BG), β-D-cellobiohydrolase (CBH), β-1,4-N-acetylglucosaminidase (NAG), L-leucine aminopeptidase (LAP), and acid phosphatase (AP) were measured using the microplate fluorescence method [60].

Microbial nutrient limitation status was assessed using unimodal enzymatic stoichiometry, which calculates the enzyme activity vector length (VL) and angle (VA). The vector length (VL) reflects the degree of carbon limitation, while the vector angle (VA) distinguishes nitrogen and phosphorus limitations: VA > 45° indicates phosphorus limitation, and VA < 45° indicates nitrogen limitation. The greater the deviation from 45°, the stronger the corresponding nitrogen or phosphorus limitation [61]. The calculation formulas for VL and VA are as follows:

$$\mathrm{VL}\;=\sqrt{{\left[\frac{\mathrm{LN}\left(\mathrm{BG}+\mathrm{CBH}\right)}{\mathrm{LN}\left(\mathrm{ACP}\right)}\right]}^{2}+{\left[\frac{\mathrm{LN}\left(\mathrm{BG}+\mathrm{CBH}\right)}{\mathrm{LN}\left(\mathrm{NAG}+\mathrm{LAP}\right)}\right]}^{2}}$$

$$\mathrm{VA}\;({}^{\circ})=\mathrm{DEGREES}[\mathrm{ATAN}2(\frac{\mathrm{LN}\left(\mathrm{BG}+\mathrm{CBH}\right)}{\mathrm{LN}\left(\mathrm{ACP}\right)},\frac{\mathrm{LN}\left(\mathrm{BG}+\mathrm{CBH}\right)}{\mathrm{LN}\left(\mathrm{NAG}+\mathrm{LAP}\right)})]\;\;$$

#### 4.3.3. Rhizosphere Soil DNA Extraction and High-Throughput Sequencing

DNA extraction from soil samples, PCR amplification, library preparation, and amplicon sequencing were performed by Shanghai Tianhao Biotechnology Co., Ltd (Huaxian County, Shaanxi, China). Total microbial DNA was extracted from each soil sample using the E.Z.N.A. Soil DNA Kit (Omega Bio-tek, Inc., Norcross, GA, USA), and DNA quantity and integrity were assessed using a Qubit 3.0 Fluorometer (Thermo Fisher Scientific, Waltham, MA, USA) and 1% agarose gel electrophoresis. Qualified DNA was stored at −20 °C until use. The V3–V4 region of the bacterial 16S rRNA gene was amplified using primers 338F (5′-ACTCCTACGGGAGGCAGCAG-3′) and 806R (5′-GGACTACHVGGGTWTCTAAT-3′), and the fungal ITS region was amplified using primers ITS1F (5′-CTTGGTCATTTAGAGGAAGTAA-3′) and ITS2R (5′-GCTGCGTTCTTCATCGATGC-3′). Sample-specific 8 bp barcodes were incorporated into primers to enable demultiplexing. PCR products were purified, quantified, and pooled at equimolar concentrations. The pooled library was purified by gel excision and sequenced using paired-end chemistry (2 × 250 bp) on an Illumina NovaSeq platform.

#### 4.3.4. Biological Information Analysis

Raw reads were processed in QIIME 2. After demultiplexing, sequences were quality-filtered and denoised using the DADA2 plugin to infer amplicon sequence variants (ASVs) and construct the ASV feature table; chimera removal was performed during denoising. Representative ASV sequences were taxonomically classified using the RDP Classifier with a 0.70 confidence threshold, and taxonomic profiles were summarized across multiple ranks (Kingdom, Phylum, Class, Order, Family, Genus, and Species). In this study, “pathogen-associated taxa” refer to taxonomic groups that have been frequently linked in the literature to soil-borne diseases of chili pepper, including bacterial wilt and root rot. Given the taxonomic resolution limits of amplicon sequencing, taxa were primarily classified at the genus level to capture relative shifts in pathogen-associated risk, rather than to infer pathogenicity for all members within a genus. Specifically, *Ralstonia* and *Fusarium* were annotated at the genus level and are discussed as pathogen-associated taxa based on their frequent associations with bacterial wilt and root rot of chili pepper reported in previous studies. The ASV table was normalized across samples prior to diversity analysis. Alpha diversity indices were calculated in R using the “vegan” package. The bacterial functions were classified using the plant beneficial bacteria (PBB) database, which is constructed based on microbial taxonomy and functional traits [40]. The primary lifestyles of fungi were classified using the Fungal Traits tool database [26].

#### 4.3.5. Network Analysis and Functional Annotation

Microbial co-occurrence networks for each treatment were independently constructed using rarefied ASV tables. Microbial network matrices were built in R (version 4.5.1) using the “reshape2” and “WGCNA” packages, implemented in RStudio Desktop (https://posit.co/download/rstudio-desktop/, accessed on 23 January 2026). [62,63], and network visualization was generated in Gephi 0.10.1 (edges with a Spearman correlation coefficient |r| > 0.8, *p* < 0.01 were retained). To evaluate the complexity of the microbial network and the interspecies interaction relationships, we calculated the network features, including the number of nodes, edges, average degree, graph density, modularity index, and the proportion of positive and negative interactions within the network. These feature values were computed using the “igraph” package [64]. Subsequently, Pearson correlation analysis was employed to assess the relationships between community cohesion, crop disease incidence, and disease index.

Cohesion is an indicator for measuring microbial interactions [65]. Here, positive and negative cohesion more accurately indicate a community’s tendency toward stronger positive vs. negative association structure, which may arise from biotic interactions and/or shared (or opposing) environmental responses. When calculating each cohesion index, the relative abundance of each species in the sample was multiplied by its corresponding connectivity value, and the products of all species were summed up. This cohesion index can be calculated as follows (after excluding rare species).

$$\mathrm{Cohesion}=\sum_{i=1}^{n}{abundance}_{i}\times {connectedness}_{i}$$

For functional prediction, bacterial community functions were annotated using PICRUSt2 on the 16S rRNA gene sequences [66]. The functional gene annotation heatmap was constructed using the R package “pheatmap” Fungal metabolic pathways were annotated using the MetaCyc database [67].

#### 4.3.6. Data Analysis

To clarify the relationships among soil properties, rhizosphere microbiota, and crop performance, we applied complementary statistical approaches. Random forest analysis was employed to identify the most influential microbial taxa and functional pathways associated with yield variation because it can handle multicollinearity and non-linear relationships among high-dimensional microbial features. Mantel tests were used to evaluate the correspondence between microbial community dissimilarity (beta diversity distance matrices) and environmental/soil variables, which is appropriate for relating multivariate community patterns to physicochemical gradients. Pearson correlation was applied to quantify pairwise associations between key indicators when variables approximated normality; otherwise, rank-based correlation was used. Together, these complementary approaches allowed us to (1) screen key predictors, (2) link community structure to soil properties, and (3) verify relationships among selected indicators.

After normalizing the ASV abundances in each sample, diversity and differential analyses were performed. A random forest regression model built using the “randomForest” R package, combined with Pearson correlation analysis, was used to identify key microbial indicators influencing chili pepper yield. Mantel test, combined with Pearson correlation analysis, was used to explore the relationships between pathogen fungi, pathogen bacteria, carbon, nitrogen, phosphorus cycling functional genes, soil nutrients, microbial nutrient limitations, and chili pepper yield. The figure and tables were created using Microsoft Excel 2021, Origin 2024b, and R 4.5.1 software. Data were analyzed using variance analysis (ANOVA) with IBM SPSS Statistics 27 software, and the significance level was set at *p* < 0.05. For variables with significant differences, Duncan’s multiple range test was used for post hoc analysis, with the significance level also set at *p* < 0.05.

## 5. Conclusions

This study demonstrated that the pepper–rice rotation (RVR) effectively alleviated disease pressure and yield decline associated with continuous monocropping by improving soil nutrient availability and rhizosphere microbial functionality. The RVR treatment significantly increased available nutrients and enzyme activity, promoted the directional proliferation of beneficial functional groups, and inhibited pathogen expansion by enhancing community modularity and competitive interactions. Particularly, key fungal metabolic pathways, including galactose degradation, sulfate reduction, and L-tryptophan degradation, played pivotal roles in both disease suppression and yield promotion. These findings indicated that the paddy–upland rotation not only achieved stable and high pepper yields but also provided a practical approach for the green management of soil-borne diseases and soil health restoration. This study offered practical insights for optimizing poaceous–solanaceous crop rotation systems while highlighting rhizosphere interactions between different crops as a potential ecological foundation for alleviating continuous cropping obstacles. Future research should investigate the regulatory effects of root exudates from these two taxonomic groups on soil microbial communities and their underlying molecular mechanisms, providing novel strategies for optimizing rational poaceous–solanaceous rotation systems and advancing sustainable agriculture.

## Figures and Tables

**Table 1:** Incidence of bacterial wilt and root rot, and yield of pepper plants under different treatments in 2023–2024.

Year		Yield (kg·ha^−1^)	Bacterial Wilt Incidence Percentage (%)	Bacterial Wilt Disease Index	Root Rot Incidence Percentage (%)	Root Rot Disease Index (%)
2023	RVR	102,835 ± 602.85 ^a^	36.06 ± 0.61 ^c^	22.22 ± 0.30 ^c^	24.81 ± 2.99 ^c^	14.25 ± 1.63 ^c^
	CCV	91,933 ± 282.90 ^b^	46.28 ± 0.85 ^b^	28.33 ± 0.63 ^b^	30.62 ± 0.93 ^b^	19.72 ± 0.49 ^b^
2024	RVR	103,694 ± 347.70 ^a^	25.89 ± 0.73 ^d^	12.34 ± 0.92 ^d^	10.84 ± 1.10 ^d^	5.86 ± 0.30 ^d^
	CCV	64,379 ± 828.15 ^c^	58.49 ± 0.84 ^a^	44.08 ± 0.41 ^a^	40.91 ± 0.77 ^a^	30.36 ± 0.83 ^a^

Note: RVR: chili–rice rotation; CCV: chili continuous cropping. Data represent the mean ± standard deviation of three biological replicates. Significant differences (*p* < 0.05) between RVR and CCV for the 2023–2024 data are indicated by lowercase letters.

**Table 2:** Alpha Diversity of Rhizosphere Microbiota under RVR and CCV Treatments.

Sample ID		Chao1	Shannon	Ace	Observed ASVs
RVR	16S-V3-V4	4076.55 ± 249.28 ^a^	7.26 ± 0.19 ^a^	4068.68 ± 246.69 ^a^	4049 ± 242.95 ^a^
CCV		3868.52 ± 744.11 ^a^	6.96 ± 0.55 ^a^	3868.45 ± 744.03 ^a^	3854.33 ± 743.08 ^a^
RVR	ITS	533.67 ± 49.12 ^a^	4.57 ± 0.11 ^a^	533.67 ± 49.12 ^a^	533.67 ± 49.12 ^a^
CCV		634.33 ± 39.58 ^a^	4.46 ± 0.28 ^a^	640.72 ± 45.28 ^a^	634.33 ± 39.58 ^a^

Note: RVR: Chili–rice rotation; CCV: Chili continuous cropping. Different lowercase letters indicate significant differences between the RVR and CCV treatments for 16S and ITS (*p* < 0.05, Duncan’s multiple range test), n = 3.

## Data Availability

The datasets analysed during the current study are available in the NCBI repository (https://dataview.ncbi.nlm.nih.gov/object/PRJNA1415229 accessed on 23 January 2026).
